# Environmental DNA (eDNA) detects the invasive rusty crayfish *Orconectes rusticus* at low abundances

**DOI:** 10.1111/1365-2664.12621

**Published:** 2016-02-24

**Authors:** Matthew M. Dougherty, Eric R. Larson, Mark A. Renshaw, Crysta A. Gantz, Scott P. Egan, Daniel M. Erickson, David M. Lodge

**Affiliations:** ^1^ Department of Biological Sciences University of Notre Dame Notre Dame IN 46556 USA; ^2^ Catholic Theological Union Chicago IL 60615 USA; ^3^ Daniel P. Haerther Center for Conservation and Research John G. Shedd Aquarium Chicago IL 60605 USA; ^4^ Environmental Change Initiative University of Notre Dame South Bend IN 46617 USA; ^5^ Department of Natural Resources and Environmental Sciences University of Illinois Urbana IL 61801 USA; ^6^ Department of BioSciences Rice University Houston TX 77251 USA

**Keywords:** crayfish, detection probability, early detection, early warning, exotic species, invasive species, lake, non‐indigenous, occupancy estimation, quantitative PCR (qPCR)

## Abstract

1. Early detection is invaluable for the cost‐effective control and eradication of invasive species, yet many traditional sampling techniques are ineffective at the low population abundances found at the onset of the invasion process. Environmental DNA (eDNA) is a promising and sensitive tool for early detection of some invasive species, but its efficacy has not yet been evaluated for many taxonomic groups and habitat types.
2. We evaluated the ability of eDNA to detect the invasive rusty crayfish *Orconectes rusticus* and to reflect patterns of its relative abundance, in upper Midwest, USA, inland lakes. We paired conventional baited trapping as a measure of crayfish relative abundance with water samples for eDNA, which were analysed in the laboratory with a qPCR assay. We modelled detection probability for *O. rusticus* eDNA using relative abundance and site characteristics as covariates and also tested the relationship between eDNA copy number and *O. rusticus* relative abundance.
3. We detected *O. rusticus* eDNA in all lakes where this species was collected by trapping, down to low relative abundances, as well as in two lakes where trap catch was zero. Detection probability of *O. rusticus* eDNA was well predicted by relative abundance of this species and lake water clarity. However, there was poor correspondence between eDNA copy number and *O. rusticus* relative abundance estimated by trap catches.
4. *Synthesis and applications*. Our study demonstrates a field and laboratory protocol for eDNA monitoring of crayfish invasions, with results of statistical models that provide guidance of sampling effort and detection probabilities for researchers in other regions and systems. We propose eDNA be included as a tool in surveillance for invasive or imperilled crayfishes and other benthic arthropods.

## Abstract 2

Our study demonstrates a field and laboratory protocol for eDNA monitoring of crayfish invasions, with results of statistical models that provide guidance of sampling effort and detection probabilities for researchers in other regions and systems. We propose eDNA be included as a tool in surveillance for invasive or imperilled crayfishes and other benthic arthropods.

## Introduction

Detection of environmental DNA (hereafter eDNA) is a new and rapidly growing monitoring tool for the study and management of organisms in freshwater ecosystems (Lodge *et al*. 2012b; Rees *et al*. 2014). eDNA is the DNA extracted from an environmental sample (e.g. soil, air or water) without isolating the target organism (Ficetola *et al*. 2008); for macrobiota, an entire organism often is not present in the sample. In freshwater, the method has most frequently been applied to the detection and monitoring of invasive species (e.g. Jerde *et al*. 2011; Dejean *et al*. 2012; Goldberg *et al*. 2013; Piaggio *et al*. 2013) – although eDNA is also being applied to monitor native species of conservation concern (e.g. Pilliod *et al*. 2013). The majority of eDNA research has focused on fish (e.g. Jerde *et al*. 2011; Thomsen *et al*. 2012a) and amphibians (e.g. Ficetola *et al*. 2008; Goldberg *et al*. 2011; Dejean *et al*. 2012), but eDNA methods are being increasingly applied to a more diverse group of taxa including mammals (Foote *et al*. 2012; Thomsen *et al*. 2012b), reptiles (Piaggio *et al*. 2013), arthropods (Thomsen *et al*. 2012b), gastropods (Goldberg *et al*. 2013) and bivalves (Egan *et al*. 2015). Yet owing to the recent emergence and ongoing development of eDNA methodologies, the feasibility of this tool for many taxa and habitats still needs to be evaluated.

Invasive species are important in freshwater systems, impacting native flora and fauna (Gurevitch & Padilla 2004; Dextrase & Mandrak 2006), human resources and the economy (Lodge *et al*. 2006; Keller *et al*. 2009), and entire ecosystems (e.g. Higgins & Vander Zanden 2010). Early detection can be critically important for effective management of invasive species. Once an invasive species is widespread, its management can become infeasible and costs of control or eradication often increase exponentially (Keller *et al*. 2009; Vander Zanden *et al*. 2010). In many cases, however, early eradication is infeasible because traditional survey methods do not detect populations at low densities. Therefore, monitoring techniques that allow early detection of invaders at low densities are needed to help protect native species and ecosystems. Although a relatively new methodology, applications of eDNA have demonstrated that it can be more sensitive than traditional sampling in detecting some invasive aquatic species at low densities (Ficetola *et al*. 2008; Goldberg *et al*. 2011; Jerde *et al*. 2011; Dejean *et al*. 2012). Further, greater ease and speed of field sampling for eDNA relative to many conventional field sampling approaches could facilitate more intensive monitoring over larger landscapes than has been previously feasible.

In this study, we test the capacity for eDNA methods both to detect presence and to reflect patterns of abundance for one of the best studied invasive freshwater crayfishes (Astacoidea), the rusty crayfish *Orconectes rusticus* (Girard 1852). Several crayfish species are globally invasive, with pronounced effects on freshwater populations, communities and ecosystems (Lodge *et al*. 2012a; Twardochleb, Olden & Larson 2013). *Orconectes rusticus* has been introduced to and spread widely throughout regions like the upper Midwest of the United States (USA), where it has reduced abundance of aquatic macrophytes, invertebrates including native crayfish species, and some fishes (Wilson *et al*. 2004; Twardochleb, Olden & Larson 2013). This species continues to invade new and previously unoccupied regions (e.g. Olden, Adams & Larson 2009), and invasions by other crayfish species are ongoing in regions including Africa, Asia, the Caribbean and Europe (Lodge *et al*. 2012a). If eDNA would make earlier detection possible, then it would enable more successful and cost‐effective eradication of these crayfish invaders (Gherardi *et al*. 2011).

Only a single study to date has evaluated the use of eDNA in monitoring or detecting crayfish populations, with somewhat equivocal success (Tréguier *et al*. 2014). Crayfish may be difficult to detect through eDNA relative to organisms like fish or amphibians as a combined consequence of their exoskeletons and use of benthic (lake or stream bottom) habitats, both of which might minimize exchange of eDNA containing tissues or cells with the water column. Yet if eDNA methods can be effective in detecting crayfish and representing their abundance, then these methods hold considerable promise to improve our management of these organisms, ranging from early detection of new invasions to population and distribution monitoring of many of the world's highly imperilled crayfish species (Larson & Olden 2016).

## Materials and methods

### Study area

**Figure 1:**
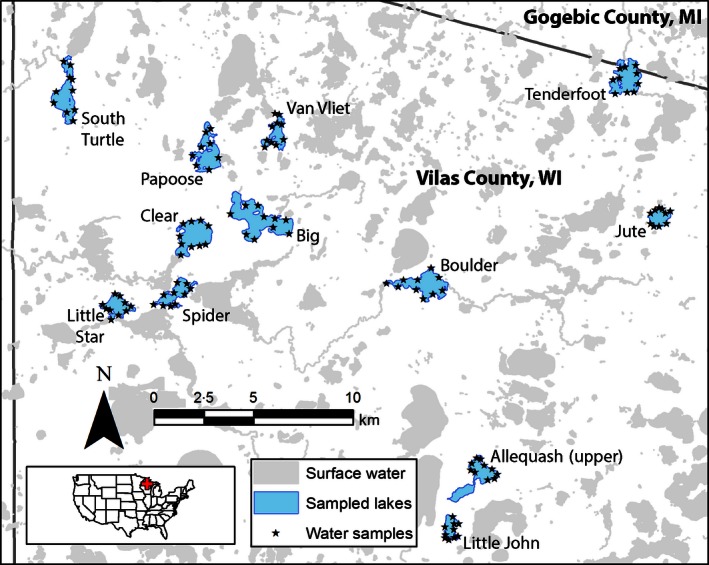
Study lakes in Vilas County, WI, and Gogebic County, MI, USA, sampled during summer 2014 for presence of *Orconectes rusticus* by both baited trapping and water samples for eDNA analyses. Locations of eDNA water samples are displayed for each lake. Locations of additional baited trap locations that did not correspond with those for water samples (see main text) are not displayed for figure clarity.

Our study was conducted in lakes of Vilas County, Wisconsin, and Gogebic County, Michigan, USA (Fig. 1). *Orconectes rusticus* has been present in this region since the 1970s following introduction through pathways including live bait releases by anglers (Capelli & Magnuson 1983; Olden *et al*. 2006). We sampled 12 lakes for both *O. rusticus* relative abundance and eDNA over two intervals during the summer of 2014: 30 July to 7 August (nine lakes) and 5–8 September (three lakes; Table 1). At each of the two intervals, lakes were sampled in sequential order from *O. rusticus* presumed absence to known high abundance, as anticipated from historic crayfish trap catch records from these lakes (e.g. Capelli & Magnuson 1983; Olsen *et al*. 1991). The sampled lakes range in size from 61 to 338 ha, with recent mean summer Secchi disc depths of 1·8–5·6 m (Table 1). Historic records (above) indicated we might also encounter the native virile crayfish *Orconectes virilis* (Hagen 1870) and previously introduced northern clearwater crayfish *Orconectes propinquus* (Girard 1852), which were included in the specificity testing during primer development for the *O. rusticus* eDNA assay.

**Table 1:** Study lakes in Vilas County, WI, and Gogebic County, MI, USA, sampled during summer 2014 with: geographic coordinates as WGS 84 latitudes and longitudes; surface areas in hectares (ha); mean summer (May–September) Secchi disc depths in metres (m) with standard deviations (SD) over the 2000–2015 time period and the number of replicates (*n*); dates of water sample collection for eDNA (earliest) and crayfish trap setting (earliest) and recovery (latest); and the number of crayfish traps recovered in each lake (in two cases, a single trap was lost or stolen). All lakes had 10 surface water samples of 250 mL taken for eDNA sampling, with the exception of Papoose lake, where only eight surface water samples were taken owing to equipment shortages. See Appendix S2 for details on sources of Secchi disc depth data and evaluation of consistency through time

Lake	Lat, Long	Area (ha)	Secchi depth (m, SD)	Secchi replicates (*n*)	Sample dates	# Traps
Tenderfoot	46·22, −89·53	177	1·8 (0·53)	4	30 July – 1 August 2014	20
Van Vliet	46·19, −89·75	89	2·7 (0·70)	60	30 July – 1 August 2014	11*
Clear	46·15, −89·81	208	3·4 (1·21)	57	2 – 3 August 2014	18
Spider	46·12, −89·82	110	3·4 (0·41)	45	2 – 3 August 2014	23*
Little Star	46·11, −89·86	99	5·6 (0·60)	27	2 – 3 August 2014	24
Boulder	46·12, −89·66	212	2·5 (0·40)	3	4 –5 August 2014	24
South Turtle	46·21, −89·90	189	2·3 (0·64)	96	4 –5 August 2014	24
Big	46·15, −89·77	338	3·2 (0·67)	117	4 –5 August 2014	20
Papoose	46·18, −89·80	173	4·5 (0·71)	44	6 –7 August 2014	24
Jute	46·15, −89·51	77	3·9 (na)	2	5 – 6 September 2014	12
Allequash	46·04, −89·62	164	2·9 (1·04)	150	5 – 6 September 2014	24
Little John	46·01, −89·65	61	2·2 (0·65)	5	6 – 7 September 2014	36

*A single trap was missing or stolen relative to historic sampling effort for these lakes (12 and 24 traps, respectively).

### Crayfish relative abundance

We estimated *O. rusticus* relative abundance using a systematic baited trapping approach that has been applied consistently in these lakes since the 1970s. Specifically, wire mesh cylindrical minnow traps with 5‐cm diameter openings were baited with approximately 120 g of beef liver and set overnight at 1–3 m depths (Capelli & Magnuson 1983). The number and location of traps set per lake was based on historic conventions for these study sites and ranged from a low of 12 traps in lakes with little habitat heterogeneity (Jute and Van Vliet lakes) to a high of 36 traps in lakes with high habitat heterogeneity (Little John Lake; Table 1). Trapped crayfish were identified to species, sexed and counted. Baited trapping is biased towards adult male *O. rusticus* over female and juvenile crayfish (Olsen *et al*. 1991). Catch per unit effort (CPUE) of only male *O. rusticus* per trap has traditionally been applied as an index of relative abundance in this system and corresponds well with direct observations of crayfish abundance in quadrats from divers (Capelli & Magnuson 1983; Olsen *et al*. 1991). We use male *O. rusticus* CPUE as an index of relative abundance in model building, but also report total CPUE for all collected crayfish species regardless of sex. See Appendix S1 in Supporting Information for more detail on trapping methodology.

### eDNA field sampling

To minimize contamination risk associated with transfer of equipment from lake to lake, we sampled lakes in sequence from *O. rusticus* absence to high abundance. We also did not collect eDNA water samples on any day that we recovered traps or handled crayfish. Further, all sampling gear was sprayed with 10% bleach after use at each lake, including the interior and exterior of the boat, traps and associated floats and lines, and equipment storage containers. Prior to sampling in the field, 250‐mL sample bottles were soaked in 10% bleach for a minimum of 10 min, rinsed and then autoclaved and stored until used in a storage container that had been wiped with bleach. At each lake except Papoose Lake, we took ten 250‐mL surface water samples matched to trap locations, which were dispersed around the lake littoral zone (Fig. 1). In Papoose Lake, we took only eight surface water samples because of an equipment shortage. Surface water samples were taken prior to trap setting to minimize risk of contamination via trap deployment to lakes, with the exception of the first two sampled lakes (Tenderfoot, Van Vliet; Table 1) where traps were set prior to surface water sampling (see Discussion). We wore nitrile gloves during water sample collection and changed gloves between each sample.

We kept water samples on ice and in a dark cooler until return to shore, where we filtered the samples immediately. We filtered samples using a hand vacuum pump (Actron CP7830; Bosch Automotive Service Solutions, Warren, MI, USA) connected to a side‐arm flask. We used filter funnels containing 1·2‐μm filters of two different materials, either cellulose nitrate (CN; first sampling interval) or polycarbonate track‐etch (PCTE; second sampling interval); filters changed between the two sampling intervals because of equipment shortage. Because sample filtering was conducted in the field (see also Goldberg *et al*. 2011), a combined cooler and filtration blank for potential contamination was taken at each lake using 250 mL of store‐bought bottled water. Filters were placed in 2‐mL microcentrifuge tubes (USA Scientific, Ocala, FL, USA) and completely submerged in 700 μL of Longmire's buffer (Longmire, Maltbie & Baker 1997). Filtered samples were stored in a refrigerator for a maximum of 8 days prior to transport to the University of Notre Dame, South Bend, IN USA, and subsequent eDNA extraction from the filters and buffer.

### Primer development

We downloaded from GenBank the cytochrome c oxidase subunit 1 (COI) sequences for the 12 species of crayfishes likely to occur in lakes in the upper Midwest, USA (Peters *et al*. 2014; Table 2), and subsequently designed primers with PrimerHunter (Duitama *et al*. 2009). Recommended primer pairs were synthesized by Integrated DNA Technologies (IDT) and evaluated in the laboratory for successful amplification with *Orconectes rusticus* DNA, both tissue‐derived and filtered aquarium water samples, as well as reduced amplification with tissue‐derived DNA from nine of the non‐target species (Table 2): *Cambarus diogenes* (Girard 1852), *Fallicambarus fodiens* (Cottle 1863), *Orconectes immunis* (Hagen 1870), *O. obscurus* (Hagen 1870), *O. propinquus* (Hobbs & Fitzpatrick 1962), *O. sanbornii* (Hobbs & Fitzpatrick 1962), *O. virilis* (Girard 1852), *Procambarus acutus* (Girard 1852) and *P. clarkii* (Girard 1852). Tissue‐derived DNA for all species tested was diluted to 1 ng μL^−1^ prior to testing in order to normalize results across species. The best primer pair, Orusticus_COI_5F (5′‐CAGGGGCGTCAGTAGATTTAGGTAT‐3′) and Orusticus_COI_5R (5′‐CATTCGATCTATAGTCATTCCCGTAG‐3′), produced a 128‐bp amplicon.

**Table 2:** Similarity of primers to common crayfish in the upper Midwest region of the USA (per Peters *et al*. 2014). We report crayfish species by scientific names, homology of the query to the forward and reverse primers described in the main text, percentage identity as a function of the number of matching base sites divided by 51 (total number of base sites across the primer pair), and the GenBank accession number of the query sequence. Base site homology between the query and the primer is shown as a dot

Crayfish Species	Forward	Reverse	Identity, %	GenBank
*Orconectes rusticus*	●●●●●●●●●●●●●●●●●●●●●●●●●	●●●●●●●●●●●●●●●●●●●●●●●●●	100	AY701249
*Orconectes obscurus*	●●●●●●●A●●●●●●●●●●●G●●●●●	T●●●●●●●●C●●●●●T●●●●●T●●●●	88	AF474355
*Orconectes sanbornii*	●T●●●●●T●●G●●●●●●●●●●●●●●	●●●●●●●●●C●●●●●T●●●●●T●●●●	88	AF474359
*Orconectes virilis*	●●●●●●●A●●●●●●●●●●●●●●●●●	T●●A●●●●●C●●●●●T●●C●●●●C●●	86	AF474365
*Orconectes propinquus*	●T●●A●●T●●●●●●A●●●●●●●●●●	T●●●●●●●●C●●●●●T●●●●●T●CG●	80	DQ889165
*Cambarus bartonii* (Fabricius 1798)	●●●●C●●●●●●●●●●●●C●T●●●●●	T●●A●●●●●C●●●●●C●●A●●G●CCA	78	AY701190
*Procambarus acutus*	●●●●T●●A●●T●●●●●●●●●●●●●●	●●●●●●G●●C●●●●●T●●A●●TACC●	78	AF474366
*Cambarus diogenes*	●●●●●●●A●●G●●●●●●●●●●●A●●	T●●A●●●●●C●●●●●T●●A●●TACT●	76	JX514472
*Fallicambarus fodiens*	●T●●C●●A●●●●●G●●●C●●●●●●●	T●●A●●●●●C●●●●●T●●●●●●ACC●	76	KC163698
*Orconectes immunis*	●T●●A●●A●●●●●C●●●●●G●●●●●	T●●●●●●●●C●●●●●T●●A●●T●CT●	76	DQ882095
*Cambarus robustus* (Girard 1852)	●G●●C●●●●●●●●●●●●C●T●●●●●	●●●A●●●●●C●●●●●T●●A●●AACCA	75	JX514491
*Procambarus clarkii*	●G●●A●●A●●T●●●●●●●●●●●●●●	T●●●●●●●●C●●G●●T●●C●●TACT●	75	AY701195

### eDNA sample processing

The eDNA extraction followed a modified chloroform–isoamyl alcohol (hereafter ‘CI’) DNA extraction and isopropanol precipitation as outlined in Renshaw *et al*. (2014): [1] the 2‐mL microcentrifuge tubes were incubated in a 65°C water bath for a minimum of 10 min; [2] 700 μL of CI (24:1, Amresco, Solon, OH, USA) was added to each tube and samples were vortexed for 5 s; [3] tubes were centrifuged at 15 000 ***g*** for 5 min and 500 μL of the aqueous layer was transferred to a fresh set of 1·5‐mL microcentrifuge tubes; [4] 500 μL of ice cold isopropyl alcohol and 250 μL of 5 m NaCl were added to the 500 μL removed from the aqueous layer and tubes were precipitated at −20 °C overnight; [5] the precipitate was pelleted by centrifugation at 15 000 ***g*** for 10 min and the liquid was decanted; [6] 150 μL of room temperature 70% ethanol was added to each tube to wash pellets; [7] tubes were centrifuged at 15 000 ***g*** for 5 min and the liquid was decanted; [8] 150 μL of room temperature 70% ethanol was added to each tube to wash pellets a second time; [9] tubes were centrifuged at 15 000 ***g*** for 5 min and the liquid was decanted; [10] pellets were dried in a vacufuge at 45 °C for 15 min, followed by air drying until no visible liquid remained; and finally, [11] pellets were rehydrated with 100 μL of 1X TE Buffer, Low EDTA (USB).

Four qPCR replicates were run for each eDNA extract in the following 20‐μL reactions: 4·85 μL of PCR‐grade water, 4 μL of 5X Colorless GoTaq^®^ Flexi Buffer (Promega, Madison, WI, USA), 0·4 μL of 10 mm dNTPs, 1·6 μL of 25 mm MgCl_2_, 1 μL of each 10‐μm primer (forward and reverse), 0·15 μL of GoTaq^®^ Flexi DNA Polymerase (Promega), 1 μL of EvaGreen (20X in water; Biotium, Hayward, CA, USA), 2 μL of 4‐μg μL^−1^ Bovine Serum Albumin (Amresco) and 4 μL of eDNA extract. Mastercycler^®^ ep Realplex (Eppendorf, Hauppauge, NY, USA) cycling conditions were as follows: an initial denaturation at 95 °C for 3 min; 45 cycles of denaturation at 95 °C for 30 s, annealing at 65 °C for 45 s and extension at 72 °C for 1 min; followed by a melting curve analysis that transitioned from 60 °C to 95 °C over a span of 20 min. To test for potential inhibition, 1:10 dilutions of each eDNA extract were run in separate qPCR assays following this protocol.

For the quantification of eDNA samples, a 500‐bp gBlock Gene Fragment (IDT) was synthesized based on GenBank accession AY701249, from base 226 to base 725. The targeted amplicon was located in the middle of the synthesized fragment, with 186 bp on either side. Copy number for the gBlock fragment was estimated by multiplying Avogadro's number by the number of moles. A serial dilution of the gBlock fragment provided a range in copy numbers for the quantification of eDNA unknowns (Gunawardana *et al*. 2014; Renshaw *et al*. 2014; Svec *et al*. 2015).

### Controls

We adopted a sequential set of controls to identify possible contamination at each step in the eDNA process (from lake to laboratory). For each set of eDNA samples (by lake), we checked the field eDNA filtration technique for contamination by filtering 250 mL of bottled water through a single filter that was then processed in the laboratory separately from the eDNA samples. In the laboratory, we checked the eDNA extraction reagents and technique for contamination by the inclusion of a single extraction (one per each set of eDNA samples) that involved just the reagents. On each plate of qPCR assays, we checked the assay reagents and technique for contamination with two wells that included the same master mix as the rest of the plate with sterile water in place of the eDNA extract. The serial dilution of standards on each plate served as a qPCR‐positive control. Finally, a single qPCR replicate from every positive eDNA amplification was confirmed through unidirectional Sanger sequencing with the reverse primer.

### Statistical analyses

We used hierarchical occupancy estimation models (MacKenzie *et al*. 2002) to investigate detection probabilities for *O. rusticus* eDNA (Schmidt *et al*. 2013). Observing occupancy (psi) of an organism at a given location is determined not only by occupancy itself, but also by the ability to detect the organism when present (detection probability; *p*). Failure to detect an organism at an occupied site is referred to as a ‘false negative’ and considerable effort has been expended over recent years to develop models that can quantify the prevalence of, and correct for, such false negatives. This is accomplished by hierarchical models that infer not only occupancy but also detection probability, as estimated from sampling at sites that is replicated or repeated in either space or time. These methods have only recently been proposed for and applied to eDNA studies (Schmidt *et al*. 2013), where they offer considerable promise in quantifying detection probabilities from eDNA sampling and providing guidance on the number of samples necessary to detect a species (or its eDNA) when present.

We used the two‐level occupancy model of MacKenzie *et al*. (2002) to model detection probability of *O. rusticus* eDNA with the *unmarked* library in version 3.1.2 of the statistics program r (R Development Core Team 2009). Lakes were our unit of occupancy, and eDNA water samples were our replicated units for estimating detection probability (Schmidt *et al*. 2013). Due to our relatively small sample size (12 lakes) and known high prevalence of *O. rusticus* in this region, we did not model occupancy itself with any covariates (Schmidt *et al*. 2013), but instead sought to characterize detection probability of *O. rusticus* eDNA using three covariates: the relative abundance of this species as estimated by baited trapping (male CPUE), lake area and mean summer Secchi disc depth (Table 1; Appendix S2).

We expected that detection probability of *O. rusticus* eDNA would improve with increasing *O. rusticus* relative abundance, and we sought to characterize thresholds of *O. rusticus* relative abundance where detection was feasible with eDNA (i.e. this approach is only useful for early warning of new invasions if detection is possible at low densities). Lake area was included with the expectation that *O. rusticus* eDNA might be more difficult to detect in larger habitats. Secchi disc depth was included as a measure of water clarity with respect to two potential effects on eDNA detection: clearer water would allow greater UV penetration that might degrade eDNA and shorten its persistence in the environment; alternatively, less clear water might contain substances (e.g. humic acid) that could inhibit qPCR in the laboratory and reduce detection probability (Rees *et al*. 2014; Jane *et al*. 2015). Finally, we estimated the number of water samples necessary to produce a cumulative 95% probability of detecting *O. rusticus* eDNA when actually present based on our most supported model using McArdle's (1990) cumulative probability equation for detecting rare species (Schmidt *et al*. 2013).

We related average eDNA copy number (log + 1 transformed) from four qPCR replicates to the same three covariates used above in occupancy modelling via multiple linear regression models for both lake averages and individual paired trap and water sample locations, and for this latter analysis also performed hierarchical mixed effects models where lake identities were included as random effects (*lme4* library, R Development Core Team 2009). Our primary interest was in evaluating if eDNA copy number corresponded with observed crayfish relative abundance, while accounting for potentially confounding covariates or other differences between lakes (i.e. random effects). Model comparisons for both occupancy (above) and abundance (eDNA copy number) analyses were made using the modified Akaike Information Criterion (AICc) for small sample sizes, where the best supported model was identified by the lowest AICc value. Models compared to this best supported model were considered equivalent at ΔAICc <2 (Burnham & Anderson 2002).

## Results

Our primer pair exhibited complete to greatly delayed reduction in amplification for all nine non‐target species evaluated (Table 2). Only non‐target *O. virilis* amplified, but with a difference (delay) in approximately 17·5 cycles relative to *O. rusticus*. Further, *O. rusticus* DNA was not detected in any of the eDNA field or laboratory negative controls. Amplification efficiencies for qPCR assays (based on the slope of the standard curve) ranged from 0·95 to 1·05 for all plates assayed for this study.

We collected *O. rusticus* in nine of 12 sampled lakes, with a range of male CPUE where trapped of 0·08 to 10·80, over the intended sequence of *O. rusticus* absence or low abundance to high abundance (Table 3). We found the non‐target crayfish species *O. propinquus* and *O. virilis* in two and four lakes, respectively, at low relative abundances (maximum total CPUE of 0·17 and 0·58, respectively). *Orconectes rusticus* eDNA was detected in 11 of 12 lakes, including two lakes (Allequash, Tenderfoot) where the species was not collected by trapping. Subsequent Sanger sequencing of eDNA samples from all positive lakes confirmed eDNA as *O. rusticus* per comparison to GenBank sequence data. Finally, where *O. rusticus* eDNA was found, copy numbers were generally low, but infrequently very high (Table 3).

**Table 3:** Results of field (baited trapping) and eDNA sampling for rusty crayfish *Orconectes rusticus* in study lakes in Vilas County, WI, and Gogebic County, MI, USA, sampled during summer 2014. Catch per unit effort (CPUE) for *O. rusticus* is given as males (male) and both sexes combined (total), and both sexes combined (total) for the non‐target species *Orconectes propinquus* and *Orconectes virilis*, with standard deviations (SD). Positive detections of *O. rusticus* eDNA by qPCR are given as proportions of water samples, as well as the average eDNA copy number from samples by qPCR with standard deviations (SD). Lakes are ordered by sampling date and sequence (see Table 1)

Lake	Crayfish Catch per Unit Effort (CPUE; # per trap)			
	Male (SD), Total *O. rusticus* (SD)	Total *O. propinquus* (SD)*, O. virilis* (SD)	eDNA Detections	Average eDNA copy number (SD)
Tenderfoot	0·00 (0·00), 0·00 (0·00)	0·15 (0·37), 0·00 (0·00)	1/10 (0·10)	0·061 (0·193)
Van Vliet	0·08 (0·00), 0·17 (0·40)	0·00 (0·00), 0·58 (0·50)	1/10 (0·10)	0·003 (0·008)
Clear	0·11 (0·32), 0·22 (0·54)	0·00 (0·00), 0·00 (0·00)	1/10 (0·10)	0·081 (0·257)
Spider	1·52 (1·77), 1·83 (2·07)	0·00 (0·00), 0·04 (0·20)	6/10 (0·60)	7·089 (18·686)
Little Star	1·71 (1·99), 1·83 (2·12)	0·00 (0·00), 0·00 (0·00)	9/10 (0·90)	2·750 (3·910)
Boulder	5·67 (6·19), 7·29 (7·52)	0·00 (0·00), 0·13 (0·61)	8/10 (0·80)	3·809 (5·614)
South Turtle	7·83 (8·96), 8·25 (9·24)	0·17 (0·38), 0·13 (0·45)	9/10 (0·90)	330·231 (1036·274)
Big	10·80 (11·81), 13·35 (13·58)	0·00 (0·00), 0·00 (0·00)	7/10 (0·70)	2·896 (5·030)
Papoose	10·42 (6·88), 11·42 (7·33)	0·00 (0·00), 0·00 (0·00)	8/8 (1·00)	2·540 (2·488)
Jute	0·00 (0·00), 0·00 (0·00)	0·00 (0·00), 0·00 (0·00)	0/10 (0·00)	0·000 (0·000)
Allequash	0·00 (0·00), 0·00 (0·00)	0·00 (0·00), 0·00 (0·00)	1/10 (0·10)	0·086 (2·640)
Little John	8·49 (7·02), 8·86 (7·03)	0·00 (0·00), 0·00 (0·00)	7/10 (0·70)	1·604 (2·643)

### Detection probability

**Figure 2:**
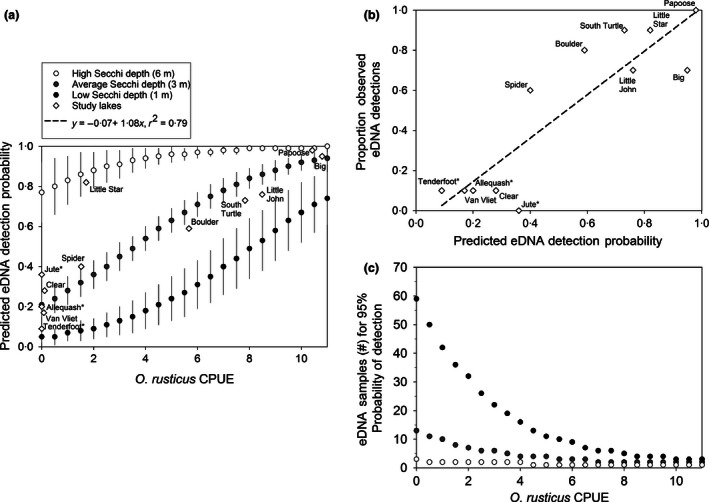
Results of occupancy estimation models accounting for detection probability for *Orconectes rusticus* based on frequency of detection in eDNA samples (Table 3). (a) Predicted detection probability for *O. rusticus* (assuming the species is present) on gradients of male catch per unit effort (CPUE) and Secchi disc depth based on the most supported model (Table 4), along with predicted eDNA detection probabilities for the 12 study lakes (Table 1; Fig. 1). (b) The proportion of observed eDNA detections from field sampling in study lakes (Table 3) plotted against predicted eDNA detection probability using observed *O. rusticus* CPUE and Secchi disc depths and based on the most supported model (Table 4). Fit of observed field detections to predicted detection probabilities is given with a linear regression model. For both (a) and (b), those lakes where no *O. rusticus* were collected by baited trapping are indicated with an asterix (*; Table 3). (c) The predicted number of eDNA samples necessary for a cumulative 95% detection probability for *O. rusticus* as predicted from male CPUE and Secchi disc depth from the most supported model (Table 4).

Our best supported model of *O. rusticus* detection used male CPUE and Secchi disc depth as covariates; no other model was within ΔAICc <2 (Table 4). Lake area had little effect on detection of *O. rusticus* eDNA. Detection probabilities increased with increasing male CPUE and also increased with increasing water clarity (Fig. 2; Table 4). Observed proportions of eDNA detections in the field were closely matched by predicted detection probabilities from the best supported model, with an *r* ^2^ = 0·79 from a linear regression model (Fig. 2). We estimated that *O. rusticus* can be detected when present at a cumulative probability of 95% with low sampling effort when this species occurs at moderate‐to‐high abundances irrespective of water clarity (Fig. 2). Alternatively, where *O. rusticus* occurs at lower densities or relative abundances, our best supported model predicts that effort to detect this species at a cumulative probability of 95% varies with water clarity. Only 2–3 water samples may be sufficient to detect *O. rusticus* in clear lakes (6‐m Secchi disc values), whereas up to 60 water samples per lake might be necessary to detect low relative abundance of *O. rusticus* in lakes with very low (1 m) Secchi disc values (Fig. 2).

**Table 4:** Model specifications (psi is occupancy and p is detection probability), parameter estimates with standard errors (SE), comparisons between models by ΔAICc and Akaike weights (wAICc) for all models considered in occupancy estimation of the crayfish *Orconectes rusticus* based on frequency of eDNA detections from water samples (see Table 3). Occupancy was not modelled by any covariates

Model	Intercept (SE)	*O. rusticus* CPUE (SE)	Secchi depth (SE)	Lake area (SE)	ΔAICc	wAICc
psi(.)p(CPUE+Secchi)	−3·88 (0·97)	0·37 (0·07)	0·85 (0·27)	–	0·00	0·99
psi(.)p(CPUE+Secchi+area)	−2·95 (1·16)	0·40 (0·07)	0·83 (0·29)	−0·01 (0·00)	4·04	0·01
psi(.)p(CPUE)	−1·19 (0·50)	0·34 (0·08)	–	–	7·85	0·00
psi(.)p(CPUE+area)	0·07 (0·66)	0·39 (0·08)	–	−0·01 (0·00)	8·61	0·00
psi(.)p(Secchi)	−1·83 (0·70)	–	0·65 (0·22)	−	34·11	0·00
psi(.)p(Secchi+area)	−2·14 (0·84)	–	0·65 (0·22)	0·02 (0·00)	38·37	0·00
psi(.)p(.)	0·18 (0·19)	–	–	–	40·74	0·00
psi(.)p(area)	−0·03 (0·48)	–	–	0·00 (0·00)	44·17	0·00

### eDNA copy number and relative abundance

**Figure 3:**
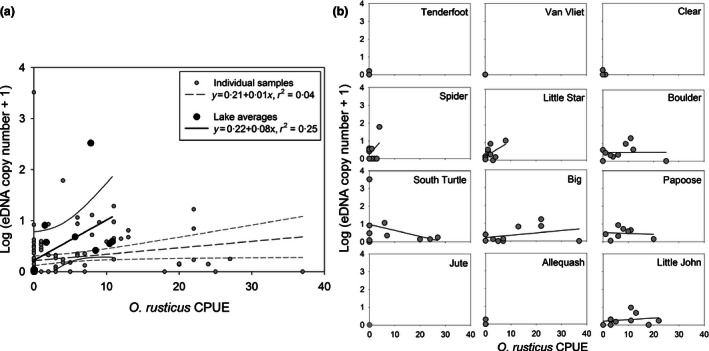
Relationships between *Orconectes rusticus* relative abundance as measured by male catch per unit effort (CPUE) from baited trapping and *O. rusticus* eDNA copy number in water samples, for both mean values of lakes and individual samples from all lakes combined (a) and individual samples within lakes (b), with fit provided by linear regression models. Copy number of eDNA is log + 1 transformed (Table 3). Whole lake data are plotted and modelled as both lake averages for male CPUE and eDNA copy number, as well as results for every individual paired water sample and baited trap (see main text), with 95% confidence intervals.

eDNA copy number was best predicted by models that only included *O. rusticus* relative abundance (male CPUE); models including lake area and/or Secchi disc depth were less supported, with all ΔAICc ≥4·69 for multiple regression models on lake average values and all ΔAICc ≥5·59 for hierarchical mixed effect models. Multiple linear regression models on individual trap and water sample pairs were more supported than the hierarchical mixed effects models on the same data that accounted for potential differences between lakes as random effects (all ΔAICc >6·64); accordingly, the linear regression model on *O. rusticus* male CPUE is reported in Fig. 3, but supplemented with plots and regressions for each individual lake. Linear regression models on lake average eDNA copy number and male CPUE explained more variation than models on individual trap and water sample pairs, but this relationship was still noisy (Fig. 3).

## Discussion

Our study demonstrates that eDNA is a viable monitoring tool for early warning of crayfish invasions. We detected *O. rusticus* eDNA in all lakes where the species was collected by baited trapping down to low observed relative abundance (0·08 male CPUE), and further detected *O. rusticus* eDNA in two lakes where the species was not directly collected. We believe that these two incidents represent greater sensitivity of eDNA to *O. rusticus* presence than conventional sampling rather than contamination, because we did not observe contamination in any of our controls. However, although the frequency (and modelled probability) of detection of *O. rusticus* eDNA increased with increasing relative abundance of this species, eDNA copy number in water samples had poor correspondence to *O. rusticus* relative abundance. As such, eDNA copy number in samples may not be adequate to represent crayfish population size or precisely reflect population trends in space or time. Further refinements in eDNA sample collection and laboratory techniques may improve on this result, but the present ability to detect invasive crayfishes at low densities via eDNA is a valuable advancement, especially for the management of these organisms.

We detected *O. rusticus* eDNA in two lakes where we did not collect this crayfish by baited trapping, and also where it has never been observed historically (Capelli & Magnuson 1983; Olsen *et al*. 1991; D.M. Lodge unpublished data). Under such circumstances, eDNA is typically commended for greater sensitivity to organism presence than conventional sampling (e.g. Jerde *et al*. 2011; Dejean *et al*. 2012; Schmidt *et al*. 2013), but an alternative interpretation is that these incidents represent ‘false positives’ as a potential consequence of field or laboratory contamination, or the presence of transported eDNA in the absence of the organism itself. None of our controls on either lake showed evidence of contamination; however, in at least one of these cases (Tenderfoot Lake), setting of crayfish traps prior to surface water sampling could have contributed to potential contamination. Alternatively, both lakes where *O. rusticus* eDNA was detected without trap catch confirmation have downstream surface water connections to systems invaded by *O. rusticus*: Tenderfoot Lake drains into the invaded Ontonagan River (Bobeldyk & Lamberti 2008), whereas Allequash Lake drains into invaded Trout Lake (Wilson *et al*. 2004). As a consequence, we believe it is possible that *O. rusticus* occurs at low abundances in these lakes, has perhaps introduced its mtDNA into these lakes via previously observed asymmetrical hybridization of *O. rusticus* females with *O. propinquus* males (Perry *et al*. 2001), or the DNA of *O. rusticus* could have been transported into these lakes by adjoining waters (Deiner & Altermatt 2014).

Our models estimated that detection of *O. rusticus* eDNA was affected not only by the relative abundance of this species, but also by lake water clarity, where detection probability improved in clearer lakes. This result suggests that UV penetration into the water column (and resultant degradation of DNA) was not an important factor in eDNA availability in our field samples, although our somewhat restricted range of values for Secchi disc depths does not exclude this being an issue in clearer lakes. Alternatively, our results suggest some substances contributing to lower lake water clarity may potentially inhibit PCR amplification (Rees *et al*. 2014; Jane *et al*. 2015). As one possible example, many lakes of our study region have brown or stained water owing to high dissolved organic carbon (DOC) concentrations (Beisner, Dent & Carpenter 2003), and DOC may result in humic acid inhibition (Jane *et al*. 2015). Our dilution of the original DNA extracts, which is a common method to reduce inhibition, did not produce any significant increase in estimates of copy number. Further research is needed to clarify the mechanism behind this pattern or identify whether it is an artefact of our relatively small sample size (8–10 samples per lake, 12 lakes total).

eDNA copy number in water samples failed to correspond well with male CPUE of *O. rusticus* at either the lake average or individual trap levels. It is perhaps not surprising that catch of crayfish at an individual trap does not correlate with eDNA copy number from surface water sampled directly above the trap; crayfish can move considerable distances to recruit to baited traps in lakes (Larson & Olden 2016), and eDNA might circulate widely in lakes owing to wave action and currents. The modest agreement between average male CPUE and eDNA copy number we observed at the whole lake scale might be improved upon through a number of means, including taking larger volumes of water for eDNA samples to reduce random variation inherent in smaller sample volumes, or investigating whether benthic rather than surface water samples improve agreement between eDNA copy number and crayfish relative abundance.

Previously, Tréguier *et al*. (2014) investigated the ability of eDNA to detect the presence of the invasive crayfish *P. clarkii* in small ponds in France. In contrast to our results, Tréguier *et al*. (2014) found that eDNA only detected *P. clarkii* in 59% of ponds where this species was trapped, whereas we detected *O. rusticus* eDNA in all lakes where this species was physically collected. Divergent results between our two studies might be the consequence of different field and laboratory methodologies. We took larger volume water samples (10 × 250 mL per lake against 6 × 15 mL per pond in Tréguier *et al*. (2014)), potentially increasing the probability of capturing DNA in our study. In addition, Tréguier *et al*. (2014) sampled from the bottom of ponds after disturbing surface sediments to resuspend benthic eDNA; while eDNA concentration is likely to be higher in sediments than in overlying water (even for pelagic fishes), sediments are also likely to cause substantial inhibition in the detection of eDNA (Turner, Uy & Everhart 2015). We collected water from the surface of lakes, potentially avoiding these inhibition issues. Tréguier *et al*. 2014 also sampled a larger number (158) of smaller (0·0007–0·8951 hectares) ponds; accordingly, eDNA false negatives may have been more prevalent in Tréguier *et al*. (2014) owing to higher replication of smaller habitats that could be more intensively sampled for crayfish by conventional methods than our larger lakes. Finally, our different results in the sensitivity of eDNA to detect invasive crayfishes may represent inherent differences between study systems and organisms, which may only be resolved as eDNA continues to be evaluated for more habitat types and taxonomic groups.

### Management implications

Invasive crayfishes have had severe negative effects on native freshwater species and ecosystems (Twardochleb, Olden & Larson 2013), and new crayfish invasions are reported every year from across the globe (Lodge *et al*. 2012a). Well‐established populations of invasive crayfishes are difficult to control or eradicate, requiring high effort and cost from managers (Gherardi *et al*. 2011). Early detection facilitates more effective control and eradication of invasive species (Vander Zanden *et al*. 2010), and our study demonstrates that eDNA is a viable tool for surveillance of new crayfish invasions at the low population sizes where management is most tractable. We anticipate that our findings will apply to monitoring of other crayfishes, such as the ecologically similar signal crayfish *Pacifastacus leniusculus* (Dana 1852), which commonly invades temperate lakes in regions including Europe, Japan and western North America (Lodge *et al*. 2012a). Indeed, our success with eDNA for crayfish suggests that this tool may be useful for other benthic arthropods.

Environmental DNA methods may therefore be a useful addition to monitoring programs for early warning of new invasions and secondary spread of benthic arthropods, similar to how eDNA has been applied for surveillance of other freshwater invasive taxa (Rees *et al*. 2014). As a specific example, eDNA could be used to monitor for crayfish invasions above barriers constructed to protect upstream populations of highly imperilled native crayfishes, providing early warnings if these management interventions have failed or been breached (Frings *et al*. 2013). Furthermore, a large proportion of native crayfishes are globally imperilled with extinction (Richman *et al*. 2013) and often occur at low abundances in difficult to sample environments (Larson & Olden 2016). Our study demonstrates that eDNA has high potential for monitoring trends in distributions and occupancy for both invasive and imperilled benthic arthropods.

## Data accessibility

Data are available from Dryad Digital Repository doi:10.5061/dryad.3hp15 (Dougherty *et al*. 2016).

## Supporting information


**Appendix S1.** Additional detail on baited trapping for crayfish.

**Appendix S2.** Secchi disc depth data, trends of lake clarity over time, and sensitivity test of model dependence of Secchi disc values.
