# Supplementary material for: Environmental DNA (eDNA) detects the invasive rusty crayfish Orconectes rusticus at low abundances
Source: J Appl Ecol. 2016 Feb 24;53(3):722–32. doi: 10.1111/1365-2664.12621 (PMC5053277; doi:10.1111/1365-2664.12621)
Supplement: Supplementary file 1 — Appendix S1. Additional detail on baited trapping for crayfish. [file JPE-53-722-s001.pdf]

Online Supporting Information for "Environmental DNA (eDNA) detects the invasive rusty crayfish (Orconectes rusticus) at low abundances."

Appendix S1. Additional detail on baited trapping for crayfish

We estimated *O. rusticus* relative abundance in study lakes for comparison to eDNA sampling by using a systematic baited trapping approach that has been applied consistently in this region (Vilas County, WI and Gogebic County, MI, USA) since 1972, beginning with Capelli & Magnuson (1983) and continuing with studies including Lodge, Kratz & Capelli (1986), Olsen *et al.* (1991), Garvey *et al.* (2003), Wilson *et al.* (2004), and Peters & Lodge (2013). Adherence to the general trapping protocol of Capelli & Magnuson (1983) over time has facilitated long-term study of population dynamics and ecosystem effects of *O. rusticus* in this intensively-researched lake district (Carpenter *et al.* 2007), making it among the best-studied crayfish invasions in the world (Lodge *et al.* 2012; Twardochleb, Larson & Olden 2013). No methodology for field sampling of crayfishes is perfect or without biases (reviewed extensively in Larson & Olden in press), but the baited trapping protocol of Capelli & Magnuson (1983) has compared favorably over time to independent estimates of crayfish relative abundance based on visual surveys by divers (Olsen et al. 1991). Accordingly, we have high confidence in the ability of this baited trapping protocol to reflect patterns of relative abundance of *O. rusticus* in our lakes, but report here some additional aspects of this baited trapping protocol for interested readers and to facilitate replicability in, and comparison to, other study systems.

First, life history stage or timing can affect crayfish catch by baited traps (Larson & Olden in press). Baited trapping for *O. rusticus* in our study region is generally timed for a July-September intermolt interval when the majority of *O. rusticus* males are in the Form I reproductively active stage of

cyclic dimorphism found in crayfishes of the family Cambaridae; these crayfishes alternate between a reproductively inactive (Form II) and active (Form I) stage that can be identified by differing morphology of the male gonopods (or first two pleopods). Form I male crayfish of the family Cambaridae are generally highly aggressive, and these males exclude females (and smaller juveniles) from traps, causing severe sex bias in CPUE (Larson & Olden in press). Main text Table 3 reveals the extent to which our total *O. rusticus* CPUE is dominated by males, despite a generally 1:1 sex ratio of the actual population. Catch-per-unit effort (CPUE) of only male *O. rusticus* per trap has traditionally been applied as an index of relative abundance in this system, and corresponds well with direct observations of crayfish abundance in quadrats from divers (Capelli & Magnuson 1983; Olsen *et al.* 1991). We use male *O. rusticus* CPUE as an index of relative abundance in model building (see below), but also report total CPUE for all collected crayfish species regardless of sex (main text Table 3).

Traps used in this study system are wire mesh cylindrical "Gee" minnow traps with 0.42 m long by 0.21 m diameter dimensions, an approximately 5.0 cm opening on each end of the cylinder, and 0.64 cm mesh. Traps are baited with approximately 120 g of beef liver and set overnight at 1 -3 m depths. Duration of overnight sets has not been found to affect catch-per-unit effort (CPUE) of crayfish in this system (Olsen *et al.* 1991). Crayfish have been found at considerably greater depths than 1-3 m in other systems, particularly in deep-water oligotrophic lakes (e.g., Flint 1977; Lewis 1997). Capelli & Magnuson (1983) expected that crayfish would not use deeper waters in our generally mesotrophic lakes where benthic (lake bottom) production is often constrained to the light-penetrating nearshore (or littoral) zone, and because lakes in this region generally have soft muck substrates at greater depths that are unsuitable for our target species (Hein *et al.* 2006). We suspect that our two cases of *O. rusticus* eDNA detections in lakes where this crayfish has never been collected by baited trapping do not represent consistent use of deeper water habitats by this crayfish than 1-3 m, but instead may reflect low density recent invasions with too few individuals to be detected by baited trapping. We discuss alternative

possibilities, including sample contamination, eDNA transport, and hybridization with *O. propinquus,* in the main text manuscript.

Trapping effort for lakes of Vilas and Gogebic counties is based on both lake size or shoreline length and observed habitat heterogeneity (see below); trap effort per lake increases up to 74 traps in the largest regularly monitored lake (Trout Lake at 1608 hectares), which is not included in the current study owing to poor sampling weather (high wind) on the intended sampling days (but see Lodge, Kratz & Capelli 1986; Wilson *et al.* 2004 for more detail on Trout Lake). Trap effort in our 12 study lakes ranged between 12 to 36 traps per lake, with most lakes (9) receiving similar trap effort between 18 and 24 trap sets. Traps are always set a minimum of 100 m apart (Capelli & Magnuson 1983; Olsen *et al.* 1991), a distance sufficient to ensure independence based on typical overnight recruitment distances of crayfishes to baited traps that range from 56 m^2 to 116 m^2 (Abrahamsson & Goldman 1970; Acosta & Perry 2000; reviewed in Larson & Olden in press).

Observers sampling for crayfish in our study system record substrate classifications at trap locations, and these classifications include “muck” or soft organic substrates, sand, and cobble, with all substrate classifications amended to include presence or absence of aquatic macrophytes. Different crayfish species have different preferences for and tolerances of benthic habitat types, from primary burrowing crayfishes that require soft or silty substrates (Dorn & Volin 2009) to species like *O. rusticus* that are dependent on rocky substrates for shelter from predators (Garvey *et al.* 2003). The role of substrate in structuring crayfish communities in our study region is well-documented, dating back to Capelli & Magnuson (1983). The three dominant crayfishes in our study lakes (*O. propinquus*, *O. rusticus*, and *O. virilis*) all prefer rocky or cobble habitats; however, in response to *O. rusticus* invasion, *O. propinquus* is generally extirpated owing to factors including hybridization with *O. rusticus* (Perry *et al.* 2001), whereas *O. virilis* is able to shift to less preferred habitats like aquatic macrophyte-dominated muck or sand substrates, and consequently is more likely to coexist with *O. rusticus* over time (Garvey

et al. 2003; Peters & Lodge 2013). This is consistent with results of our own 2014 baited trapping, where *O. virilis* was encountered in more lakes and at higher relative abundance than *O. propinquus* (main text Table 3).

Low trap effort (12 traps) at two lakes and high trap effort (36 traps) at one lake is based on historically observed benthic habitat heterogeneity, with lower effort lakes having fewer benthic habitat classifications along their shoreline. For example, the low sampling effort (and low *O. rusticus* abundance) Van Vliet Lake was dominated by muck substrates at an observed 7 of 12 (58%) of trap locations during summer 2014 with more favorable cobble substrates at a very low 2 of 12 (16%) trap locations, whereas the higher trapping effort (and high *O. rusticus* abundance) Little John Lake is more evenly divided between muck (11/36; 31%), sand (13/36; 36%), and cobble (12/36; 33%). Failure to trap *O. rusticus* from two lakes where eDNA of this species was detected might be attributable to trap effort, although both lakes (Allequash, Tenderfoot) received intermediate trapping effort (20-24 traps). Further, although these lakes were generally poor in *O. rusticus*-preferred rock substrates, our sampling effort still included some trap sets in suitable habitats. Five of 24 (21%) trap sets in Allequash Lake were classified as cobble, and 4 of 20 (20%) trap sets at Tenderfoot Lake were classified as cobble. Conversely, Jute Lake, where *O. rusticus* was not detected by eDNA or trapped, had 6 of 12 (50%) trap sets classified as cobble. Despite suitable substrate, *O. rusticus* are believed to be absent from Jute Lake because dissolved calcium levels are too low in this seepage lake (no inlet or outlet streams) to support crayfish (Capelli & Magnuson 1983; Olden et al. 2006).

Finally, with respect to failure to trap *O. rusticus* from both Allequash and Tenderfoot lakes, we note that both locations have been trapped by the authorship team 11 times since Capelli & Magnuson (1983) with no collection of *O. rusticus* (Unpublished data, D.M. Lodge). Allequash Lake is also monitored for crayfishes as part of the North Temperate Lakes Long Term Ecological Research project and University of Wisconsin Trout Lake Station, and no *O. rusticus* individuals have been trapped from

this lake in 39 sampling events from 1981 to 2014 with effort of 18 to 31 trap sets per night (https://lter.limnology.wisc.edu/dataset/north-temperate-lakes-lter-crayfish-abundance-1981-current). Given these historic sampling efforts, we do not believe failure to detect *O. rusticus* by baited trapping during summer 2014 in Allequash or Tenderfoot Lake is an artifact of trapping effort at a single event, although baited trapping may altogether be inadequate to detect extremely low population abundances of *O. rusticus* for which we propose eDNA is potentially a more sensitive monitoring tool.

References

Abrahamsson, S.A.A. & Goldman, C.R. (1970) Distribution, density and production of the crayfish *Pacifastacus leniusculus* Dana in Lake Tahoe, California-Nevada. *Oikos*, **21**, 83-91.

Acosta, C.A. & Perry, S.A. (2000) Effective sampling area: a quantitative method for sampling crayfish populations in freshwater marshes. *Crustaceana*, **73**, 425-431.

Capelli, G.M. & Magnuson, J.J. (1983) Morphoedaphic and biogeographic analysis of crayfish distribution in northern Wisconsin. *Journal of Crutacean Biology,* **3**, 548-564.

Carpenter, S.R., Benson, B.J., Biggs, R., Chipman, J.W., Foley, J.A, Golding, S.A., *et al.* (2007) Understanding regional change: a comparison of two lake districts. *BioScience*, **57**, 323-335.

Dorn, N.J. & Volin, J.C. (2009) Resistance of crayfish (*Procambarus* spp.) popualtions to wetland drying depends on species and substrate. *Journal of the North American Benthological Society*, **28**, 766-777.

Flint, R.W. (1977) Seasonal activity, migration and distribution of the crayfish, *Pacifastacus leniusculus*, in Lake Tahoe. *American Midland Naturalist*, **97**, 280-292.

Garvey, J.E., Rettig, J.E., Stein, R.A., Lodge, D.M., & Klosiewski, S.P. (2003) Scale-dependent associations among fish predation, littoral habitat, and distributions of crayfish species. Ecology, **84**, 3339-3348.

Hein, C.L., Roth, B.M., Ives, A.R., & Vander Zanden, M.J. (2006) Fish predation and trapping for rusty crayfish (*Orconectes rusticus*) control: a whole-lake experiment. *Canadian Journal of Fisheries and Aquatic Sciences*, **63**, 383-393.

Kreps, T.A. (2009) Scaling up: Long-term, large-scale impacts of the invasion of lakes by the invasive rusty crayfish (*Orconectes rusticus*). PhD dissertation, University of Notre Dame, South Bend, Indiana.

Larson, E.R. & Olden, J.D. (In press) Sampling techniques for crayfish. *Biology and Ecology of Crayfish* (eds M. Longshaw & P. Stebbing), CRC Press, Boca Raton.

Lewis, S.D. (1997) Life history, population dynamics, and management of signal crayfish in Lake Billy Chinook, Oregon. M.S. Thesis, Oregon State University, Corvallis, Oregon.

Lodge, D.M., Kratz, T.K., & Capelli, G.M. (1986) Long-term dynamics of three crayfish species in Trout Lake, Wisconsin. *Canadian Journal of Fisheries and Aquatic Sciences*,**43**, 993-998.

Lodge, D.M., Deines, A., Gherardi, F., Yeo, D.C.J., Arcella, T., Baldridge, A.K., Barnes, M.A., Chadderton, W.L., Feder, J.L., Gantz, C.A., Howard, G.W., Jerde, C.L., Peters, B.W., Peters, J.A., Sargent, L.W., Turner, C.R., Wittmann, M.E. & Zeng, Y. (2012) Global introductions of crayfishes: evaluating impact of species invasions on ecosystem services. *Annual Review of Ecology, Evolution, and Systematics,* **43**, 449-472.

Olden, J.D., Adams, J.W. & Larson E.R. (2009) First record of *Orconectes rusticus* (Girard, 1852) (Decapoda, Cambaridae) west of the Great Continental Divide in North America*. Crustaceana,* **82**, 1347-1351.

Olden, J.D., McCarthy, J.M., Maxted, J.T., Fetzer, W.W, & Vander Zanden, M.J. (2006) The rapid spread of rusty crayfish (*Orconectes rusticus*) with observations on native crayfish declines in Wisconsin (U.S.A.) over the past 130 years. *Biological Invasions*, **8**, 1621-1628.

Olden, J.D., Vander Zanden, M.J., & Johnson, P.T.J. (2011) Assessing ecosystem vulnerability to invasive rusty crayfish (*Orconectes rusticus*). *Ecological Applications*, **21**, 2587-2599.

Olsen, T.M., Lodge, D.M., Capelli, G.M. & Houlihan, R.J. (1991) Mechanisms of impact of an introduced crayfish (*Orconectes rusticus*) on littoral congeners, snails, and macrophytes. *Canadian Journal of Fisheries and Aquatic Sciences,* **48**, 1853-1861.

Perry, W.L., Feder, J.L., Dwyer, G. & Lodge, D.M. (2001) Hybrid zone dynamics and species replacement between *Orconectes* crayfishes in a northern Wisconsin lake. *Evolution*, **55**, 1153-1166.

Peters, J.A. & Lodge, D.M. (2013) Habitat, predation, and coexistence between invasive and native crayfishes: prioritizing lakes for invasion prevention. *Biological Invasions*, **15**, 2489-2502.

Twardochleb, L.A., Olden, J.D. & Larson, E.R. (2013) A global meta-analysis of the ecological impacts of non-native crayfish. *Freshwater Science,* **32**, 1367-1382.

Wilson, K.A., Magnuson, J.J. Lodge, D.M., Hill, A.M., Kratz, T.K., Perry, W.L., & Willis, T.V. (2004) A long-term rusty crayfish (Orconectes rusticus) invasion: dispersal patterns and community change in a north temperate lake. *Canadian Journal of Fisheries and Aquatic Sciences,* **61**, 2255-226.
