# Supplementary material for: Environmental DNA (eDNA) detects the invasive rusty crayfish Orconectes rusticus at low abundances
Source: J Appl Ecol. 2016 Feb 24;53(3):722–32. doi: 10.1111/1365-2664.12621 (PMC5053277; doi:10.1111/1365-2664.12621)
Supplement: Supplementary file 2 — Appendix S2. Secchi disc depth data, trends of lake clarity over time, and sensitivity test of model dependence of Secchi disc values. [file JPE-53-722-s002.pdf]

Online Supporting Information for “Environmental DNA (eDNA) detects the invasive rusty crayfish (*Orconectes rusticus*) at low abundances.”

Appendix S2. Secchi disk depth data, trends of lake clarity over time, and sensitivity test of model dependence on Secchi disk values

Figure S2.1. Lake water clarity trends in time – Page 3

Figure S2.2. Sensitivity test of Secchi disk data on model results – Page 5

We included Secchi disk depth as a predictor of *O. rusticus* eDNA detection probability and copy number because it could represent two potential effects of water clarity on eDNA: clearer water would allow greater UV penetration that might degrade eDNA and shorten its persistence in the environment (Pilliod *et al.* 2014), whereas less clear water might contain substances (e.g. humic acid) that could inhibit qPCR in the laboratory and reduced detection probability (Rees *et al.* 2014; Jane *et al* 2015). We did not collect Secchi disk depth on the date of water sampling for *O. rusticus* eDNA, and instead used recent summer mean Secchi disk depths to represent gradients in water clarity between our study lakes that we anticipate reflect fundamental differences in lake bathymetry, basin geomorphology, and land cover (Canfield & Bachmann 1981; Soranno *et al.* 2010; Zhang *et al.* 2012).

We used Secchi disk depth data from 2000 to 2015 collected and published by the Wisconsin Department of Natural Resources (dnr.wi.gov/lakes) and the University of Wisconsin North Temperate Lakes Long Term Ecological Research program (lter.limnology.wisc.edu). We defined summer broadly as May to September, which increased replication of data but could also increase variability in our Secchi disk depth measurements owing to seasonal variation in the transition of late spring to summer and summer to autumn (Wetzel 2001). We supplemented this historic data by taking Secchi disk depth

measurements at Boulder, Jute, Little John and Tenderfoot lakes on July 7 2015 to provide more current measures of water clarity for four of most data-poor study sites. The count of Secchi disk depth measurements for our study lakes ranged from only two values for Jute Lake up to 150 values for Allequash Lake, and mean summer Secchi disk depths ranged between 1.8 m at Tenderfoot Lake to 5.6 m at Little Star Lake (main text Table 1).

Lake water clarity can be variable within and between years (Wetzel 2001), and we sought to evaluate the magnitude of variability in water clarity for our study lakes by regressing Secchi disk depth against sample date (Fig. S2.1). Although there is variability in water clarity of our study lakes as measured by Secchi disk depths within and between years, we found that these lakes have not generally experienced any trends in water clarity over recent time (post-2000), and our most clear lakes (Little Star, Papoose) have reliably higher Secchi disk depths than our least clear lakes (Little John, South Turtle, Tenderfoot). No lakes showed significant trends through time on the basis of 95% confidence intervals (Fig. S2.1). These results are consistent with a large data synthesis in our study region (the upper Midwest of the United States) that evaluated Secchi disk depths between 1938 and 2012 for 3,251 lakes, and found that the majority of lakes had no significant change over time in summer water clarity (Lottig *et al.* 2014). 89% of lakes showed no change in water clarity, whereas only 4% showed decreasing water clarity and 7% showed increasing water clarity. Lottig *et al.* (2014) concluded that the vast majority of lakes of the upper Midwest of the United States "exhibited relatively stable (i.e., no significant long-term directional trend) water clarity with minimal inter-annual variability…" in what they believe is "…the largest dataset of citizen-monitored Secchi data to be analyzed to date…" We similarly conclude that lake clarity is generally consistent over time and between our study sites, and that gradients of low (e.g., Tenderfoot) to high (e.g., Little Star) Secchi disk depths used in our models represent reliable and consistent differences between lakes (Fig. S2.1).

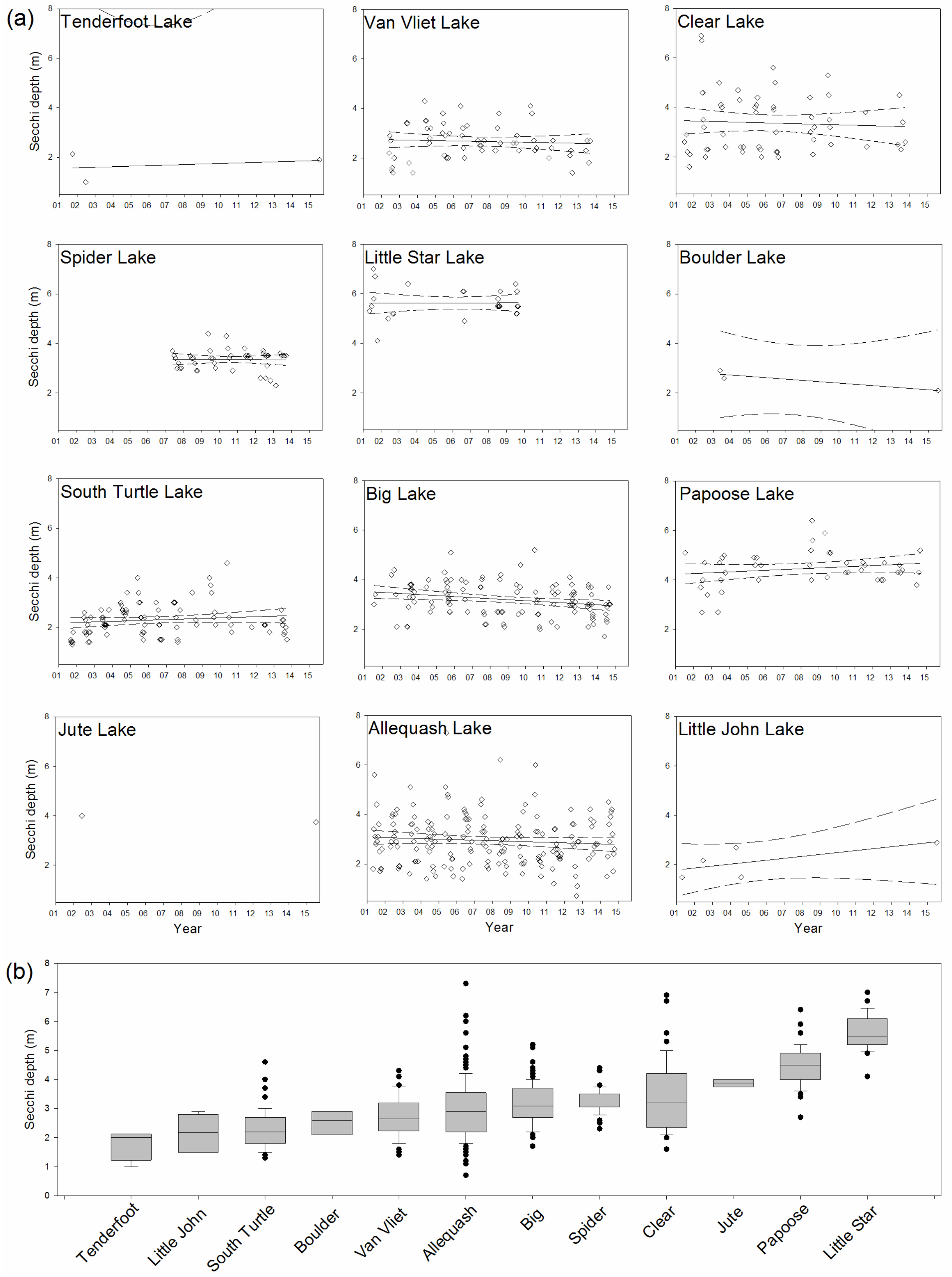
(a)
Tenderfoot Lake
Van Vliet Lake
Clear Lake
Spider Lake
Little Star Lake
Boulder Lake
South Turtle Lake
Big Lake
Papoose Lake
Jute Lake
Allequash Lake
Little John Lake
Secchi depth (m)
Year
(b)
Secchi depth (m)
Tenderfoot
Little John
South Turtle
Boulder
Van Vliet
Allequash
Big
Spider
Clear
Jute
Papoose
Little Star

Fig. S2.1. *Secchi disk depth by sample date (2000-2015) for our 12 study lakes with 95% confidence intervals (a), and box plots of all data values on an ascending gradient of lowest (Tenderfoot) to highest (Little Star) lake clarity (b).*

Despite the lack of trend in Secchi disk depth values or lake water clarity at our study sites over recent time, some variability or noise exists between and within years for these lakes. We sought to determine whether this variability within lakes exceeded that between lakes in a way that would appreciably affect results of our main text model on eDNA detection probability, for which Secchi disk depth was an important predictor (main text Table 4). Accordingly, we tested sensitivity of our main text models to the specific Secchi disk value used by randomizing 99 times from the range of Secchi disk depths observed at each individual lake (Fig. S2.1) by resampling with replacement. We then compared ΔAICc of the main text most supported model psi(.)p(CPUE+Secchi) using these randomized Secchi disk depth values to the next most supported model that did not include a Secchi disk depth, psi(.)p(CPUE). This analysis asked whether our most supported model would include Secchi disk depth over a model that included only crayfish relative abundance (CPUE) when Secchi disk depth values were randomly drawn from the range of those observed at these lakes over recent time (Fig. S2.1). We found that models including Secchi disk depth were more supported (75; 76.7%) or equally supported (19; 19.1%) to models with only crayfish relative abundance or CPUE in most cases, although there were five exceptions (5.1%) where models excluding Secchi disk depth were most supported (Fig. S2.2). For these five models, the effect of Secchi disk depth on eDNA detection probability for *O. rusticus* was still positive, but very weak. In the vast majority of cases (94.9%), models with Secchi disk depth would have been included as most or equally supported in our model results using the range of Secchi disk values

observed for our study lakes, consistent with expectations (above) that water clarity of these lakes is relatively consistent over time and variance between lakes consistently exceeds that within lakes.

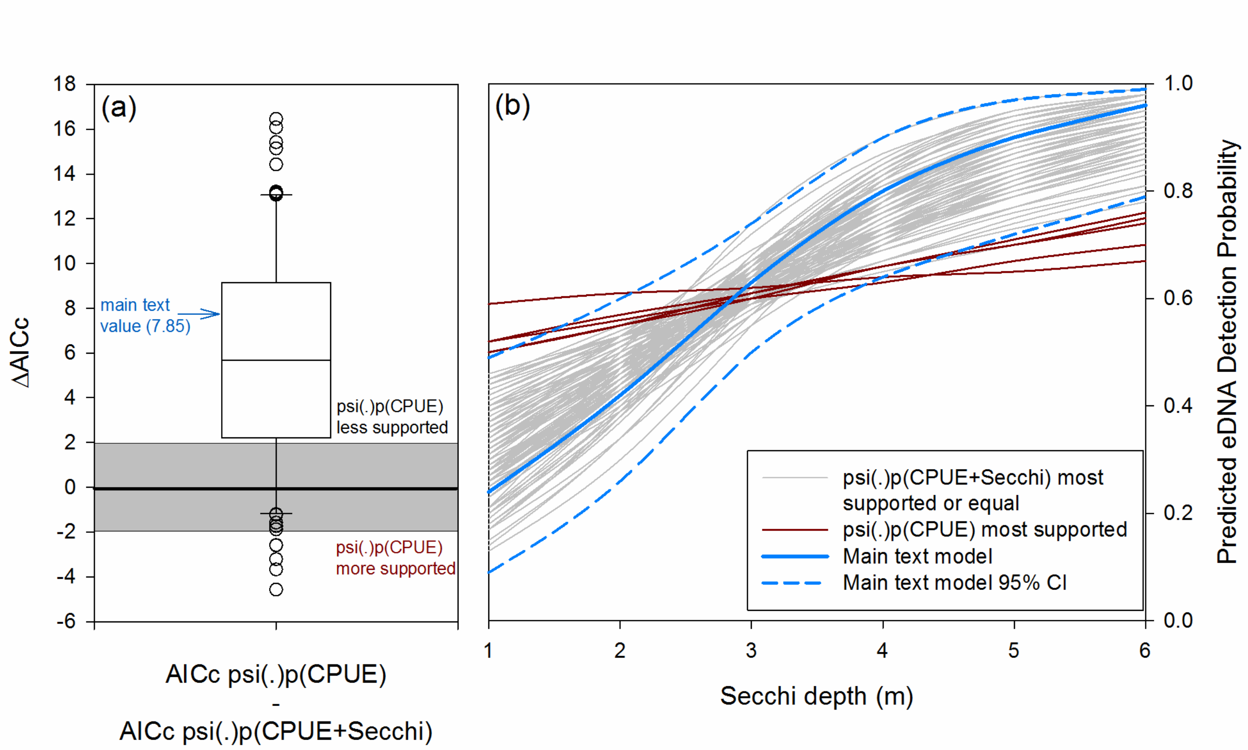

Fig. S2.2. *Comparison of ΔAICc values of the psi(.)p(CPUE) model minus the main text most supported model psi(.)p(CPUE+Secchi) for 99 randomizations of all Secchi disk depth data available for study lakes (Fig. S2.1) using resampling with replacement (a), and effect of Secchi disk depth (1-6 m) on predicted eDNA detection probability at an intermediate crayfish relative abundance of CPUE=5 for the main text model with 95% confidence intervals in blue, and data randomization models for the 94 cases where psi(.)p(CPUE+Secchi) was most supported or equally supported as psi(.)p(CPUE) in grey and the five cases where psi(.)p(CPUE) was the most supported model in red (b).*

References

Canfield, D.E., & Bachmann, R.W. (1981) Prediction of total phosphorous concentrations, Chlorophyll *a*, and Secchi depths in natural and artificial lakes. *Canadian Journal of Fisheries and Aquatic Sciences*, **38**, 414-423.

Jane, S.F., Wilcox, T.M., McKelvey, K.S., Young, M.K., Schwartz, M.K., Lowe, W.H., Letcher, B.H., & Whiteley, A.R. (2015) Distance, flow and PCR inhibition: eDNA dynamics in two headwater streams. *Molecular Ecology Resources*, **15**, 216-227.

Lottig, N.R., Wagner, T., Norton Henry, E., Cheruvelil, S. K., Webster, K.E., Downing, J.A., & Stow, C.A. (2014) Long-term citizen-collected data reveal geographical patterns and temporal trends in lake water clarity. *Plos One*, **,** e95769.

Pilliod, D.S., Goldberg, C.S., Arkle, R.S., & Waits, L.P. (2014) Factors influencing detection of eDNA from a stream-dwelling amphibian. *Molecular Ecology Resources*, **14**, 109-116.

Rees, H.C., Maddison, B.C., Middleditch, D.J., Patmore, J.R.M. & Grough, K.C. (2014) The detection of aquatic animal species using environmental DNA- a review of eDNA as a survey tool in ecology. *Journal of Applied Ecology,* **51**, 1450-1459.

Sorano, P.A., Cherevelil, K.S., Webster, K.E., Bremigan, M.T., Wagner, T. & Stow, C.A. (2010) Using landscape limnology to classify freshwater ecosystems for multi-ecosystem management and conservation. *BioScience*, **60**, 440-454.

Wetzel, R.G. (2001) *Limnology: Lake and river ecosystems.* 3rd ed. New York: Academic Press. 1006 p.

Zhang, T., Soranno, P.A., Cheruvelil, K.S., Kramer, D.B., Bremigan, M.T., & Ligmann-Zielinska, A. (2012) Evaluating the effects of upstream lakes and wetlands on lake phosphorus concentrations using a spatially-explicit model. *Landscape Ecology*, **27**, 1015-1030.
